# Strain Rate Effect on the Ductile Brittle Transition in Grinding Hot Pressed SiC Ceramics

**DOI:** 10.3390/mi11060545

**Published:** 2020-05-27

**Authors:** Pai Huang, Jiaqi Zhang

**Affiliations:** 1 Key Laboratory of Automobile Materials, Ministry of Education, College of Materials Science and Engineering, Jilin University, Changchun 130012, China; ph7771@outlook.com; 2 School of Chemistry and Environmental Engineering, Changchun University of Science and Technology, Changchun 130013, China

**Keywords:** strain rate, single-grit grinding, ductile to brittle, dislocations, amorphous

## Abstract

Surface and subsurface damage are still persistent technical challenges for the abrasive machining hot pressed-silicon carbide (HP-SiC) ceramics. Therefore, an investigation of the material behavior and critical depth of ductile to brittle transition (DBT) is essential for improving high precision and quality grinding HP-SiC ceramics. In this paper, single-grit grinding experiments with different scratch speed were conducted to study strain rate effect on the critical depth of DBT. The nanoindentations were performed to test the hardness and Young’s modulus changes of DBT position under different scratch speeds. The material removal mechanism and phase changes underneath the scratch groove were investigated using Raman tests. Based on the specific energies consumed in ductile and brittle modes of machining, a theoretical model of the critical depth of DBT was developed. The experimental results suggest that high scratch speeds generate high nanohardness, high Young‘s modulus and high critical depth of DBT of HP-SiC ceramics. The measured critical depth of DBT shows a good agreement with the predicted value calculated by the developed model. The subsurface damage depth reduced with high strain rate. Furthermore, the Raman results revealed that dislocations and amorphous transformation dominated the ductile removal mechanism of HP-SiC grinding. The fracture chips and subsurface damage depth was determined by the lateral crack and median crack, respectively. This paper’s results provide a fundamental understanding of the effect of grinding speed on the material removal mode of HP-SiC ceramics.

## 1. Introduction

Due to high specific stiffness, high chemical inertness, high thermal conductivity and enhanced radiation stability, silicon carbide (SiC) is emerging as a prime candidate for several engineering applications, which are common in combustion environments, military-grade vehicle control sensing and space exploration [1,2,3,4]. However, as a typical hard and brittleness material, hot pressed (HP)-SiC is difficult to machine as it always generating surface defects and considerable subsurface damage during processing, which affects the lifetime of components. High quality products of ceramics free of cracks can be achieved by ductile regime machining. Generally, grinding is considered to be the most effective surface finish process for obtaining a surface of high integrity and high-dimensional accuracy for ceramics [5,6]. However, the grinding process involves a complex behavior of interaction between mass random grains that are distributed on the wheel and workpiece surface. Such cases that lead to the exploration of machining deformation and removal mechanisms are difficult.

Therefore, numerous researchers adopted scratch tests in terms of experiments and simulation methods which could compare to the grinding process in order to study the removal behavior of ceramics. Cao et al. [7] found the cutting ability of the tool was significantly improved by the assistance of ultrasonic when comparing the results of scratch tests with and ultrasonic assistance. Furthermore, they put forward that the critical depth of cut for the ductile to brittle transition is increased by 56.25% with ultrasonic assistance. Similarly, Zhu et al. [8] revealed that SiC was dominated by the ductile removal mode when maximum undeformed chip thickness was below 0.29 μm in single-grit simulation. The microstructure effect of α-SiAlON was also explored using scratch tests [9]. They found the fine equiaxed microstructure exhibited high resistance to material removal at low loads. Ghatu and Raka [10] claimed that the scratch process-induced damage is closely related to the inherent porosity and the initial microstructure of ceramics. Furthermore, there are some studies that have addressed the deformation mechanism of SiC ceramics. The propagation and interaction between cracks are the main types responsible for material peeling, and scratch depth and separation of adjacent scratches dictate the interaction of cracks [11]. Meng et al. [12] and Li et al. [13,14] reveled that phase transformation and dislocation movement dominate the ductile removal of 6H-SiC and reaction-bonded SiC ceramics by completing a Transmission Electron Microscopy (TEM) test of the subsurface damaged by scratches. In the molecular dynamic (MD) simulation, Xiao et al. [15] point out that Frank partial dislocations and basal plane edge dislocations are the primary mechanism for the ductile deformation of 6H-SiC. While some fundamental understanding has been investigated, previous studies rarely considered the effect of strain rate caused by grinding speed on the critical depth of DBT. In addition, structure characterizes HP-SiCs including 3C, 4H and 6H crystal, and the way in which sinter-additives lead the relationship between material removal behavior and mechanism is complicated and not clear.

In this study, single-grit scratch tests were used to elucidate the strain rate effect on the DBT of HP-SiC. A comprehensive scanning electron microscope (SEM) observation for scratch topography and Raman analysis of the removal mechanism were completed to explain the strain rate effect on the DBT of HP-SiC ceramics. The hardness and Young’s modulus of DBT position were measured by nanoindentation. A theoretical model was developed based on the specific cutting energies consumed in ductile and brittle modes taking the scratch speed into consideration. The results will provide a more practical investigation for the ductile machining of HP-SiC, which helps to minimize and avoid the induced damages in machining processes.

## 2. Materials and Methods

**Figure 1:**
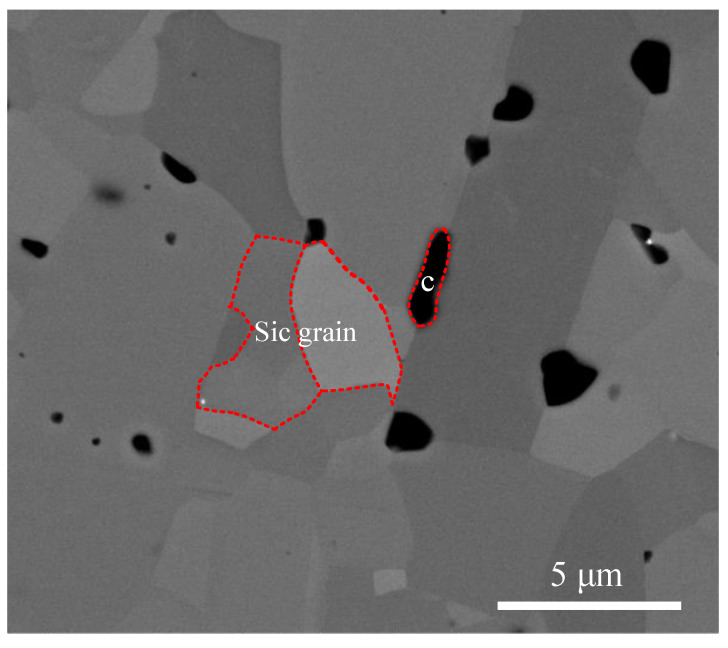
Surface topography of polished HP-SiC ceramics.

**Figure 2:**
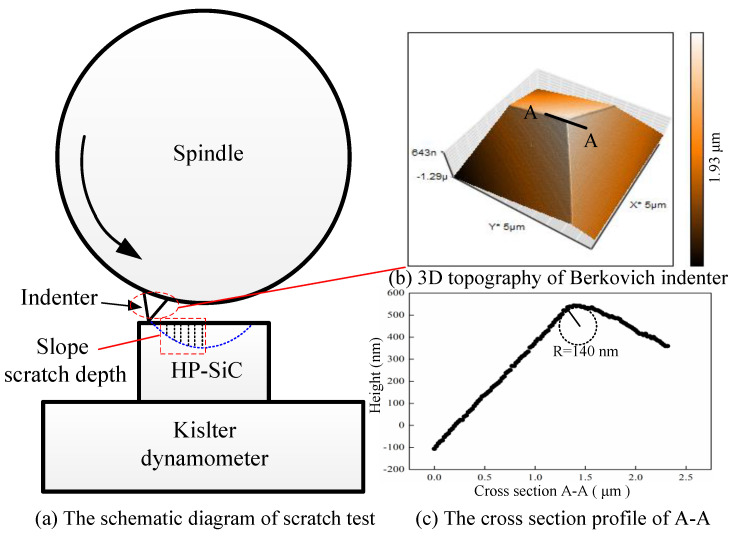
(**a**) The schematic diagram of scratch test; (**b**) 3D image of the Berkovich indenter and (**c**) cross-section profile of A–A.

The HP-SiC ceramic samples with dimensions of 10 × 10 × 5 mm used in the scratch experiments were supplied by the College of Materials Science and Engineering at Jilin University. The physical properties are listed in Table 1. To minimize surface damage effect, polishing was completed on both sides of the workpiece until the average surface roughness Sa was 5 nm, as shown in Figure 1. It can be found that SiC particles randomly distribute with different orientation, and residual carbides disperse at grain boundary. Scratch tests were performed on a three axis ultra-precision diamond turning machines. A Berkovich indenter was fixed on the aerostatic spindle by a holder with edge-forward orientation. The scratch behavior with different speeds was driven by the spindle rotation. This type of process of varied scratch depths which change from shallow to deep and finally back to shallow can ensure that the material undergoes the ductile to brittle deformation. The SiC workpiece was installed on a Kistler dynamometer (9129AA, Kislter, Winterthur, Switzerland) which is mounted on an aerostatic bearing slide. The displacement of the indenter was recorded by the displacement sensor. The procedure of the scratching experiment is illustrated in Figure 2.

To gain insight on the removal behavior corresponding to the different stain rate, the scratch speeds were chosen as 0.01 m/s, 0.3 m/s, 1 m/s and 2 m/s. The distance between each scratch trajectory was set to 2 mm to avoid the interference. In addition, to gain statistically reliable data, each scratch parameter was repeated five times and the averages were taken as the final values. All tests were carried out at room temperature. After scratch experiments, the topography of the scratch grooves were observed by SEM (Helios Nanolab 600i, FEI, Hillsboro, OR, USA). Raman spectrum were collected from chips with ductile and fracture types to uncover the deformation and fracture patterns. The critical depth of DBT was measured by atomic force microscopes (AFM). Afterwards, the nanoindentation experiments were performed at 50 mN loading using the MTS NanoXP Nanoindenter system (MTS Cooperation, Nano Instruments, Eden Prairie, MN, USA). The indenter tip is also a Berkovich indenter with a tip radius of about 60 nm. Thus, the nanohardness (H) and Young’s modulus (E) measures were deliberately made at the vicinity of, inside, and across the scratch grooves at the DBT position with z 10 μm interval. The loading and unloading times were held constant at 10 seconds. Finally, the corresponding cross sectional was polished by focused ion beam (FIB) in situ etching (Helios Nanolab 600i, FEI) and investigated by SEM.

## 3. Modelling of DBT

### 3.1. Specific Cutting Energy in the Ductile Regime

In the plastic deformation stage, the energy consumption is equal to the product of tangential force and tangential velocity. The total tangential force can be divided into three parts, namely, friction force *F_tf_*, tangential cutting chip formation force *F_tc_* and elastic restoring force *F_et_* at the rake face and flank face of the indenter, respectively.

(1) $$F_{total}=F_{tc}+F_{tf}+F_{et}$$

A previous study [16] revealed that the scratch normal load *F* can be expressed by the horizontally projected area of the contact area between the indenter and sample *A_T_*:

(2) $$F=P_{m}\cdot A_{T}$$

where *P_m_* is a contact stress that is always simplified to an average pressure *σ_y_*. In this paper, it is considered to be equal to the yield strength of HP-SiC ceramics. The yielding of a crystal is a thermally activated process and the yield stress σ_y_ can be expressed by an exponential function of the strain rate $\dot{\varepsilon }$ [17]:

(3) $$\sigma_{y}=A{\dot{\varepsilon }}^{1/n}\exp(\Delta H_{\tau }/k_{B}T)$$

where *A* and *n* are constants, and △*H_τ_* such that *n*△*H_τ_* is an energy parameter for the activation of energy for dislocation glide △*H_d_* (see, e.g., [18]). *T* is the temperature of loading that equals room temperature. The strain rate $\dot{\varepsilon }$ is the time derivative of strain, which describe the deformation velocity of the material during the scratch process. It can be expressed as follows [19]:

(4) $$\dot{\varepsilon }=\frac{v_{s}}{d}$$

**Figure 3:**
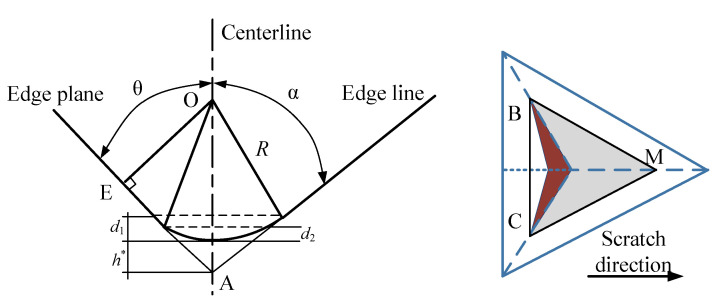
The dimension of the Berkovich indenter and projection area with an edge-forward scratch.

Furthermore, the tangential force in the ductile regime can be obtained by substituting Equation (3) and Equation (4) into Equation (2):

(5) $$F_{tc}=\sigma_{y}\cdot A_{T}$$

(6) $$F_{tf}=\mu F_{N}=\mu \sigma_{y}\cdot A_{N}$$

(7) $$F_{te}=\sigma_{y}\cdot A_{Ne}$$

where *A_T_* and *A_N_* are the projection area of the Berkovich indenter in contact with material in the vertical direction. As shown in Figure 3, the projection area can be divided into three parts based on scratch depth *d*, namely the sphere section when 0 < *d* ≤ *d*_2_, the intermediate transition section when *d*_2_ < *d* ≤ *d*_1_ and the triangular pyramid section when *d* ≥ *d*_1_. According to the geometric of Berkovich, *d*_1_ and *d*_2_ can be expressed as follows:

(8) $$d_{1}=R(1-\sin\alpha )$$

(9) $$h^{*}=R(1/\sin\alpha -1)$$

(10) $$d_{2}\cdot (d_{2}-2R+\tan\theta )=\tan\theta \cdot R\cdot (1-\sin\alpha )$$

where α is the centerline of the ridge equal to 77° and θ is the centerline of the face equal to 65.3°. *d*_1_’s calculated value is 3.58 nm base on the tip radius of the indenter measured in Section 2. In this paper, both the critical DBT depth and scratch depth are large than *d*_1_. Therefore, only the third parts should be considered. The project area of *A_N_*, *A_T_* and *A_Ne_* can be obtained by:

(11) $$A_{N}=\sqrt{3}/2\cdot (d+d_{1})\cdot \tan^{2}\beta \cdot (d+d_{1}+d_{e})$$

(12) $$A_{T}=\sqrt{3}\cdot (d-d_{e})\cdot (d+d_{e}+2d_{1})\cdot \sin\theta /\cos^{2}\theta$$

(13) $$A_{Ne}=3\sqrt{3}\cdot (d_{e}+d_{1})\cdot d_{e}\cdot \sin^{2}\theta /\cos\theta$$

where *d_e_* is depth caused by elastic recovery.

According to the reference, the elastic recovery depth can be given by the function of material hardness, elastic modulus *E* and tool geometry [20]:

(14) $$d_{e}=\lambda RH_{s}/E$$

where *H_S_* is the scratch hardness, which can be expressed by [18]:

(15) $$H_{s}=c{\left(v_{s}/d\right)}^{m}$$

where *c* and *m* are constant, *v*_s_ is the scratch velocity and *d* is the scratch groove width. Thus, finally the unit cutting energy can be obtained as:

(16) $$E_{p}=F_{total}/A_{T}=(F_{tc}+F_{tf}+F_{te})/A_{T}$$

### 3.2. Specific Cutting Energy in the Brittle Regime

**Figure 4:**
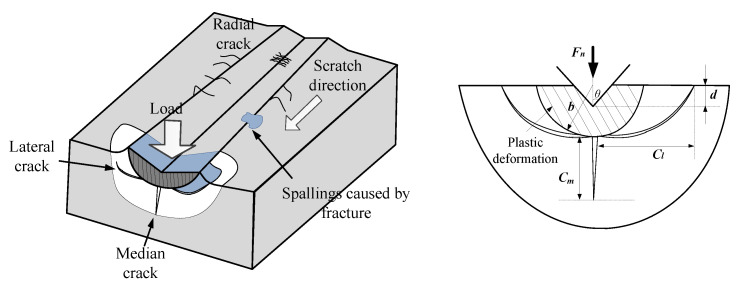
The schematic of scratch damage of the subsurface.

In the brittle fracture regime (Figure 4), with the overall consideration of the material properties, abrasive geometry and depth of cut, the length of median cracks is given by Reference [21]:

(17) $$C_{m}=C_{2}\frac{{\left(E\cdot H\right)}^{1/3}}{{\left(K_{c}\mu \right)}^{2/3}}{\left(\cot\theta \right)}^{4/9}{\left(h\cdot \tan\theta \right)}^{4/3}$$

where *C*_2_ is a constant that equals 0.206. *K*_C_ is the fracture toughness and μ is the material parameter determined by elastic recovery. For SiC, μ is 0.34.

Furthermore, according to the research by Arif et al. [22], the lateral cracks is proportional to median cracks, which can be obtained by:

(18) $$C_{l}≅\eta C_{m}$$

where *C_l_* is the lateral cracks and *η* is equal to 7 [22]. Integrating the length of median cracks in Equation (16) allows us to obtain the relation of lateral crack lengths *C_l_* to the depth of cut *h*. In addition, based on the brittle material fracture mechanics, the fracture energy *E_f_* can be expressed by the function of the fracture surface and surface energy *γ*_s_ [23]:

(19) $$E_{f}=A_{s}\gamma_{s}$$

Base on this assumption, the total fracture energy can be calculated by adding one median crack and two lateral cracks, as shown in Figure 4:

(20) $$E_{f}=(C_{l}+2C_{m})v\gamma_{s}$$

The total deformation energy underneath the fracture stage including both plastic deformation and brittle fracture energy:

(21) $$E_{b}=(E_{f}+E_{p})/V_{b}$$

(22) $$V_{b}=\frac{\pi C_{l}^{2}L}{2}$$

in which *V_b_* is the removal stock by the fracture, *L* is the scratch length per unit time, *L* = *v*.

**Figure 5:**
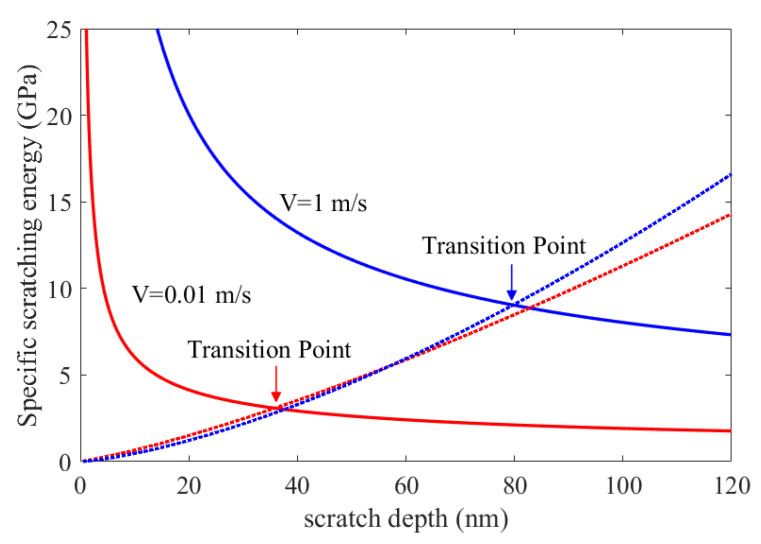
Predictions of various specific scratching energies with different scratch speeds in scratches on HP-SiC ceramics.

The energies cost in the ductile and brittle regimes were analytically developed, with consideration given to the effect of strain rate caused by scratch speed. In order to predict the effect of strain rate on the critical depth of DBT and to ensure the scratch depth could cover all whole deformation stages, the scratch depth was set to 0 to 120 nm in the simulation. Model predictions for the effect of the strain rate on the DBT are presented in Figure 5. The interaction point of the two different specific cutting energy modes is regarded as the transition point of ductile to brittle, which means that one mode of energy transitions into the other mode. It was found that the critical depth increased with the increase in scratch velocity.

## 4. Results and Discussion

### 4.1. The Residual Topography of the Scratch Groove and the Scratch Force

**Figure 6:**
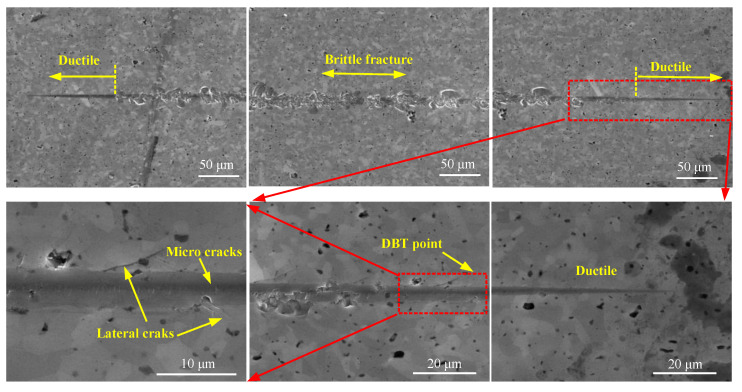
The scratch topography of HP-SiC ceramics with 2 m/s scratch speed.

**Figure 7:**
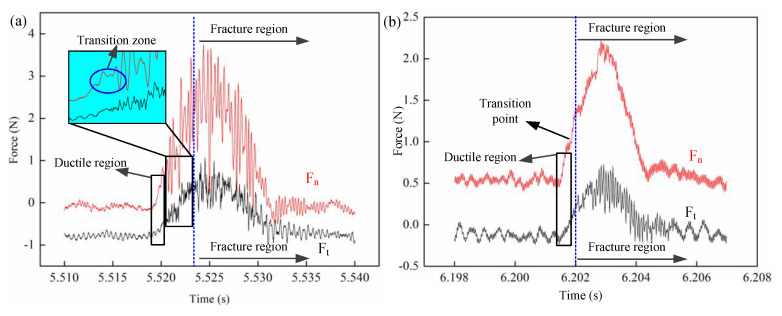
The measured force signal with the different scratch speeds; (**a**) 2 m/s; (**b**) 0.01 m/s.

Figure 6 and Figure 7 shows the residual topography of the scratch groove under the scratch velocity of 2 m/s, and the corresponding force signal. Three distinct zones can be identified in the whole scratch process according to the emergence of cracks, i.e., the ductile mode, transitional mode and brittle mode. At the first stage when the Berkovich indenter initially cut into the material, the residual scratch groove surface had no cracks and the groove width increased steadily with the increase in scratch depth. This implies that elastic and plastic deformation dominate the machining process at such shallow scratch depths. Furthermore, the corresponding scratch force increased smoothly during this period. In the following stage when the normal force and tangential force reached 0.542 and 0.363 N, respectively, microcracks emerged first at the bottom of the scratch groove. The scratch force exhibited obvious fluctuation as marked in Figure 7a, which suggests that the mode of machining transition into the transition phase before reaching the entirely brittle mode. As the critical depth of DBT is reached, cracks form and propagate at the sides of the scratch groove, and the corresponding force becomes unstable until the end of the scratching process. Note that the propagated lateral cracks caused the removed material to form microscopic chips. Figure 7a indicates. The occurrence of brittle-mode scratching as it becomes very unstable with continuous and remarkable fluctuations.

**Figure 8:**
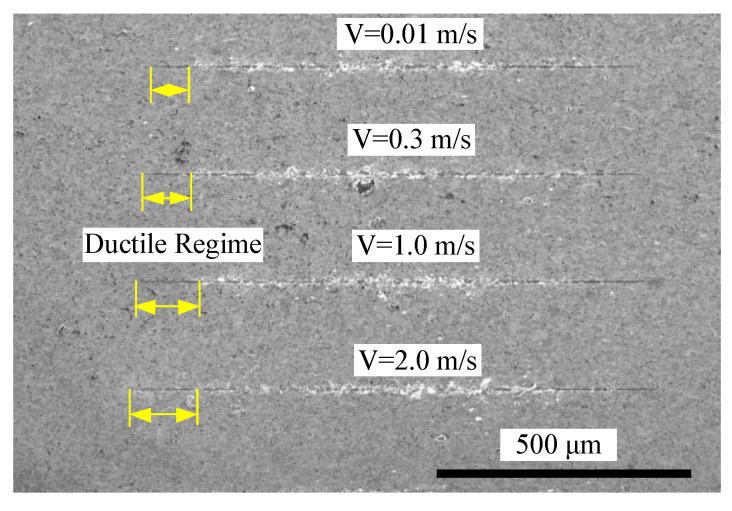
The residual scratch topography at different scratch speeds.

**Figure 9:**
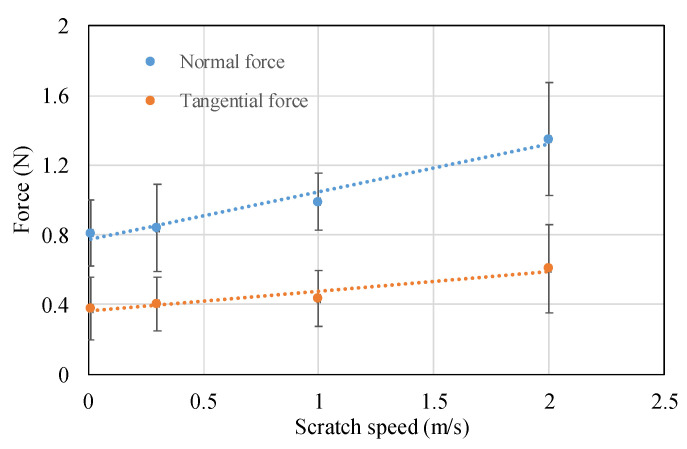
The measured force signal of DBT at different scratch speeds.

In addition, Figure 8 shows the typical scratch topography with varied scratch speeds of 0.01 m/s, 0.3 m/s, 1 m/s, 2 m/s. There is no significant difference in terms of deformation regime, crack formation and propagation, apart from the length of the ductile regime. This is because the depth of DBT change was caused by the increase in the scratch (this will be discussed in detail in Section 4.3), as each scratch trajectory has the same circumference. In contrast, it was found that the tangential and normal force at the transition point were 0.35 N and 0.48 N at 0.01 m/s scratch speed, which is much lower than 0.82 N and 0.56 N at 2 m/s scratch speed as shown in Figure 9. This phenomenon is closely related to the material deformation mechanism. Generally, the force increases with an increase in scratch speed. This finding means that the ductile energy consumption is higher in 2 m/s than 0.01 m/s, which indicates that the hardness of material increases due to high scratch speed, and that the stress for crack formation also increased.

### 4.2. Structural Analysis by Raman

**Figure 10:**
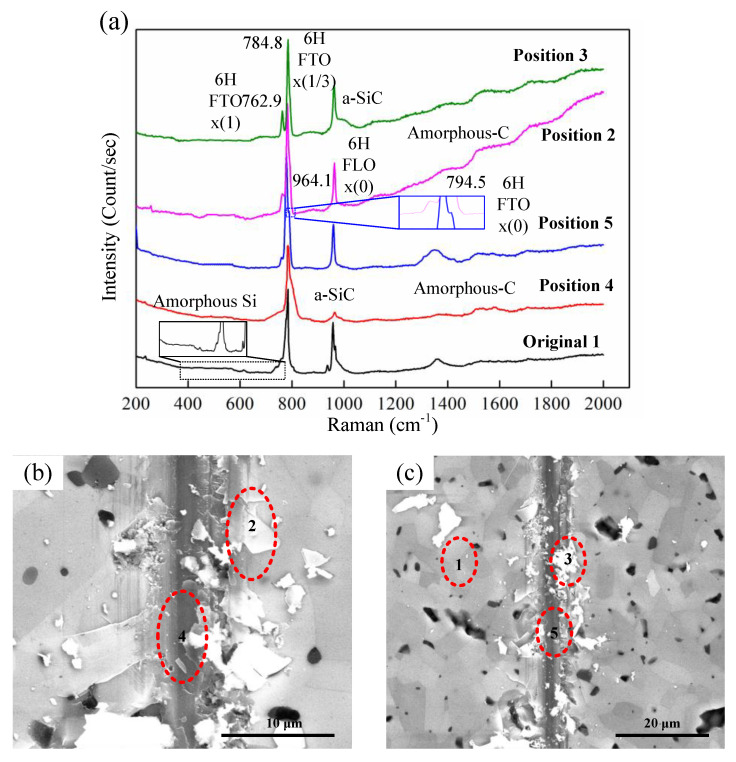
(**a**) Raman spectra collected from different positions as marked in (**b**) and (**c**); (**b**) and (**c**) Scanning electron microscope (SEM) images of topography of scratch grooves with 2 m/s scratch speed.

To reveal the reason for the strain rate effect on the critical depth of DBT of HP-SiC ceramics, the removal mechanism of material should be investigated in-depth. Raman spectroscopy is a powerful technique that is used to characterise the structure of SiC [24,25]. Figure 10a shows the Raman spectrum that was collected from the different positions that marked in Figure 10b,c. As a comparison, the Raman spectrum of the HP-SiC sample (Position 1) before being scratched is also exhibited. The spectra obtained before being scratched shows a series of Raman bands from 200 to 2000 cm^−1^ and good consistency with the spectra recorded in previously [26]. The HP-SiC is mainly composed of SiC with hexagonal close-packed (HCP) structures and residual carbides. The peaks that appear at 782.8 cm^−1^ and 758.5 cm^−1^ should be attributed to the folded modes of transverse optical (FTO) of 6H-SiC at a reduced momentum of *x* = 1/3 and 1. In the high region of 900–1000 cm^−1^, namely the longitudinal optical (LO) phonon zone, the peaks at 944.5 cm^−1^ and 966.5 cm^−1^ should be assigned to a FLO mode of 6H-SiC with *x* = 1/3 and *x* = 0. Furthermore, it should be noted that the weak emergences of a Si peak (Si–Si) at 520 cm^−1^ with a broadened band implies amorphous Si exist in original HP-SiC ceramics [27]. As seen in Figure 10a (Position 2 to 4), it is quite clear that the scratch zone has profoundly different Raman spectra. Deformation or lattice changes of the SiC structure can be observed via Raman spectra. The results suggest that within the debris (Position 3) adhered along the sides of the scratch groove, amorphous transformation of SiC occurred, implied by the broadened band at 982 cm^−1^ Raman shift. Within the groove at Position 4, a broadened band at a Raman shift of 784.8 cm^−1^ was observed, which means that there is disordered Si–C residual in the subsurface layer. Furthermore, it was observed that via the Raman spectra collected at Position 5, a new peak appeared at 794.5 cm^−1^ Raman shift. This Raman shift belongs to the 3C–SiC or 4H–SiC, indicating stacking faults induced by the compressive stress during the scratch process [28,29]. The formation of stacking faults closely related with partial dislocations movement as a result of dissociation of a perfect dislocation during plastic deformation. It can be seen that the Raman spectra shifts to a higher frequency (from 758.5 cm^−^^1^ and 782.8 cm^−^^1^ to 762.9 cm^−^^1^ and 784.8 cm^−^^1^) after being scratched, which accounts for the compressive stress induced by plastic deformation of dislocations. Thus, it can be obtained that the ductile removal mechanism of HP-SiC ceramics is controlled by amorphous transformation and dislocation motion. On the other hand, it is interesting to note that the Raman spectra obtained from fracture chips (Position 2) caused by brittle fracture show no evidence of amorphous patterns, as illustrated in Figure 10a. These results indicate that the fracture chips are generated by the propagation of cracks or the cleavage of grains. The most dramatic changes are the appearance of high frequency bands, a broad band with a higher intensity in the range of 1300 to 1600 cm^−^^1^ in the chips [30]. Such Raman bands are associated with C–C bonds, corresponding to amorphous carbon. These results indicate that impurities such as residual carbides could act as the stress concentration site which has positive effects on crack formation.

### 4.3. The Hardness and Young’s Modulus in the Scratch

**Figure 11:**
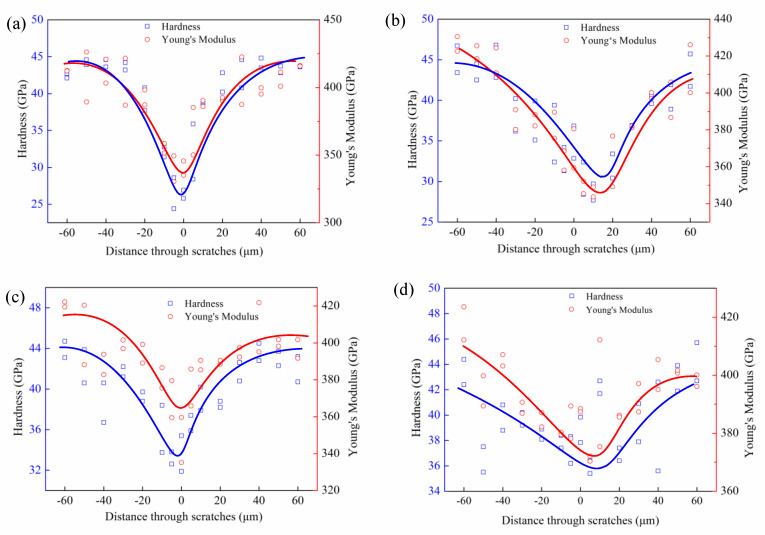
Plots of nanohardness and Young’s modulus with distance through scratches at different scratch speeds; (**a**) 0.01 m/s; (**b**) 0.3 m/s; (**c**) 1 m/s; (**d**) 2 m/s.

**Figure 12:**
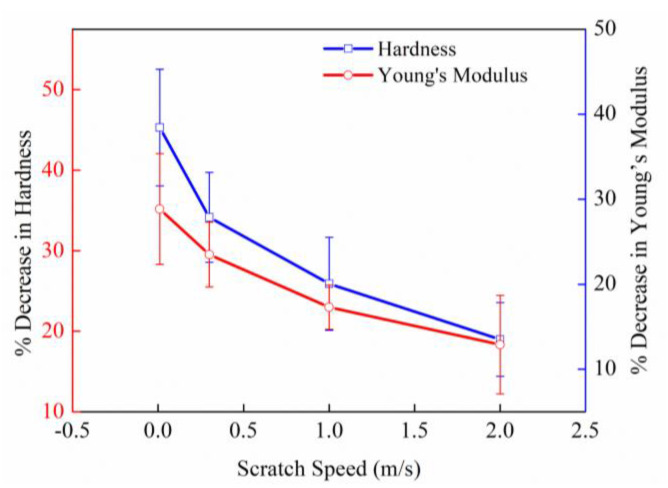
Percentage decrease in nanohardness and Young’s modulus.

For the measurement of thin film properties, nanoindentation experiments could provide a significant advantage over other mechanical testing methods. Therefore, this method was chosen to characterize the subsurface damage layer in micrometers. The data obtained from the nanoindentation experiments of nanohardness and Young’s modulus at the cross-section of the DBT position are presented in Figure 11. The minimum value always appears at the deepest points or neighboring points of the scratch grooves, then gradually increased on the both sides of the center of the groove, and finally attains the value of the maximum at locations far away from the scratch grooves. The rate of change of both nanohardness and Young’s modulus with the distance through the scratches were maximum at the middle of the scratch grooves and fell with a steep gradient, thereafter becoming constant at still further distances. This result indicates that the density of subsurface defects induced by scratching is higher at the center of the scratch grooves. On other hand, the data of maximum decrease in percentage of nanohardness and Young’s modulus changes with different scratch speeds, as shown in Figure 12. It can be seen that nanohardness depends on the scratch speed, as its value decreased by about 45.9–18.98% when scratch speed was increased from 0.01 m/s to 2 m/s, respectively. Similarly, the corresponding Young’s modulus values decreased by about 28.85% to 12.9%. This result could also explain why the depth of DBT increased with the increase in scratch speed.

### 4.4. The Depth of DBT and Subsurface Damage

**Figure 13:**
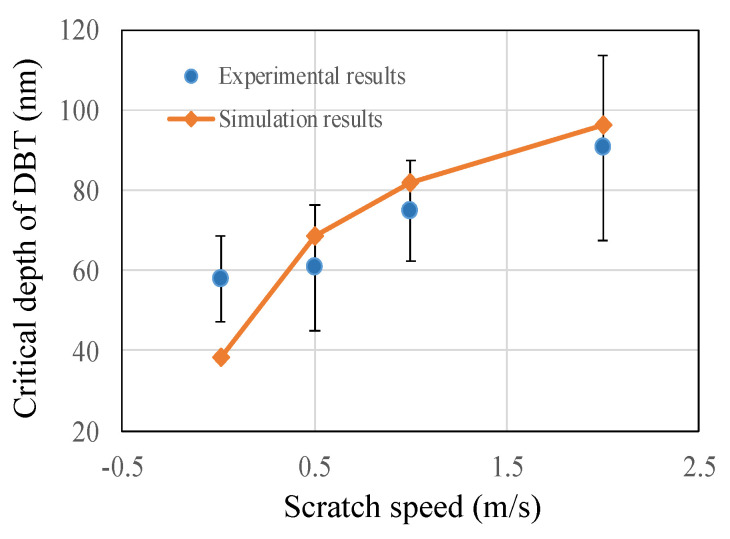
The critical depth of DBT obtained from experimental simulations.

From Raman spectra collected from beneath the scratch groove, it can be confirmed that plastic deformation of HP-SiC ceramics is governed by lattice defects (known as dislocations) and a small amount of amorphous transformation. The crystal is transported by the dislocations via bond splitting and the reforming process, and it eventually exits the grain leaving behind an atomic step in its wake. Afterwards, while the dislocations pass through each adjacent grain, the significant macroscopic strain, and hence ductility, are formed. Additionally, it is reasonable to believe that the density of dislocations and amorphous transformation are higher at high scratch speed. Afterwards, with the movement of dislocations, it will pile-up at the grain boundary and get tangled. In turn, the propagation of successive mobile dislocations is impeded. Then, the resistance of material increases, which suggests that the hardness and tensile strength increase accordingly. Consequently, the critical depth of DBT increases from 58.6 nm to 117.2 nm when scratch speed increase from 0.01 m/s to 2 m/s, as shown in Figure 13. It can also be confirmed that the estimated value based on the predicted model has a good agreement with experimental results. It is interesting to note that the predicted value is slightly bigger than the experiment results. This phenomenon might be related to defects within the HP-SiC ceramics, such as grain boundary, the carbides phase and stacking faults, as illustrated in a previous study [31].

**Figure 14:**
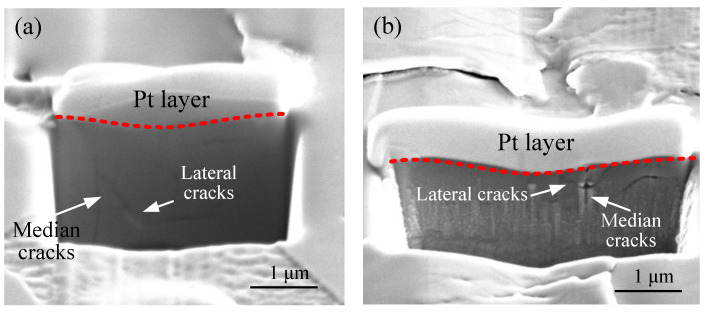
SEM images of subsurface damage (**a**) 0.01 m/s, (**b**) 2m/s.

Figure 14 shows the topography of the damaged subsurface at the DBT point of the scratch groove with 0.1 m/s and 2 m/s scratch speeds. It is obvious that higher scratch speeds lead to smaller depths of median cracks and lengths of lateral cracks. However, the crack propagation path does not conform to the regular shape and the lateral and median crack formation sites were not right underneath the contact tip between indenter and sample. This is due to the alignment of the Berkovich indenter tip and microstructural effects. This may be another reason for the error of DBT between the predicted value and experimental results. It is well known that lateral cracks occur due to residual stress when an indenter is unloaded. Thus, it can be deduced that the tensile strength HP-SiC ceramics increases with the increase in scratch speed. On the other hand, it can be observed that the extension of lateral cracks to the free surface is attributed to the material peeling, as shown in Figure 10.

## 5. Conclusions

The micro cracks and fractures are always inevitably induced in the machining of HP-SiC ceramics. The critical depth of DBT is closely related with material properties, machining conditions and complex removal mechanisms of both the ductile and brittle mode. Therefore, this paper developed a prediction model about critical depth of DBT based on the specific cutting energies consumed in the ductile and brittle modes. The effect of strain rate caused by scratch speed was taken into consideration. Then, single-grit grinding experiments were performed to verify the reliability of the model. To explore the reason for the effect of scratch speed on DBT of HP-SiC ceramics, material removal mechanisms were investigated using Raman. The following conclusions can be drawn according to the experiments and theoretical analysis:

(1) The removal characterizes of HP-SiC ceramics in a single-grit grinding process can be distinguished into three areas, i.e., ductile, DBT and fracture. A theoretical model incorporating the strain rate was developed to evaluate the scratch-induced damages.

(2) Scratch speed-dependent strain rate has an obvious effect on the critical depth of DBT of HP-SiC ceramics. An increase in scratch speed leads to a higher hardness, higher Young’s modulus and higher critical depth of DBT. The simulation value based on the proposed model agreed well with the experiments results.

(3) The ductile removal mechanism of HP-SiC ceramics is dominated by phase transformation and dislocation motions. The pile-up and tangling of dislocations decide the DBT of HP-SiC ceramics in a high strain rate.

(4) A higher scratch speed can reduce the subsurface damage depth at the DBT of HP-SiC ceramics, which suggests that a high grinding speed could inhibit the subsurface damage of HP-SiC ceramics.

## Figures and Tables

**Table 1:** Physical properties of HP-SiC.

Properties	HP-SiC
Elastic modulus E (GPa)	400
Vickers hardness H (kg/mm^−2^)	2400–2800
Shear strength (MPa)	210–380
Tensile strength (MPa)	400
Compressive strength (MPa)	1000–1700
Fracture toughness KIC (MPa·m^1/2^)	4.0–5.0
Density ρ (g/cm^3^)	3.15
Passion ratio υ	0.17
